# Snoring sound classification in patients with cerebrovascular stenosis based on an improved ConvNeXt model

**DOI:** 10.3389/fphys.2025.1661258

**Published:** 2025-11-26

**Authors:** Caijian Hua, Zhihui Liu, Liuying Li, Xia Zhou, Caorong Xiang

**Affiliations:** 1 School of Computer Science and Engineering, Sichuan University of Science and Engineering, Yibin, China; 2 Traditional Chinese Medicine Department, Zigong First People’s Hospital, Zigong, China

**Keywords:** snoring sound classification, cerebrovascular stenosis, acoustic features, ConvNeXt, dynamic convolution, attention mechanisms

## Abstract

**Introduction:**

Snoring is a common symptom of Obstructive Sleep Apnea (OSA) and has also been associated with an elevated risk of cerebrovascular disease. However, existing snoring detection studies predominantly focus on individuals with Obstructive Sleep Apnea-Hypopnea Syndrome (OSAHS), with limited attention given to the specific acoustic characteristics of patients with concomitant cerebrovascular diseases. To address this gap, this paper proposes a snoring classification method integrating dynamic convolution and attention mechanisms, and explores the acoustic feature differences between patients with cerebrovascular stenosis and those without stenosis.

**Methods:**

First, we collected nocturnal snoring sounds from 31 patients diagnosed with OSAHS, including 16 patients with cerebrovascular stenosis, and extracted four types of acoustic features: Mel spectrogram, Mel-frequency cepstral coefficients (MFCCs), Constant Q Transform (CQT) spectrogram, and Chroma Energy Normalized Statistics (CENS). Then, based on the ConvNeXt backbone, we enhanced the network by incorporating the Alterable Kernel Convolution (AKConv) module, the Convolutional Block Attention Module (CBAM), and the Conv2Former module. We conducted experiments on snoring *versus* non-snoring classification and stenotic *versus* non-stenotic snoring classification, and validated the role of each module through ablation studies. Finally, the Mann-Whitney U test was applied to compare intergroup differences in low-frequency energy ratio, snoring frequency, and snoring event duration.

**Results:**

This method achieves the best performance on the Mel spectrogram, with a snoring classification accuracy of 90.24%, compared to 88.16% for the ConvNeXt baseline model. It also maintains superiority in classifying stenotic *versus* non-stenotic snoring. Ablation analysis indicates that all three modules contribute to performance improvements. Moreover, the Mann–Whitney U test revealed significant differences (p<0.05) between the stenotic and non-stenotic groups in terms of low-frequency energy ratio and nocturnal snoring frequency, whereas snoring event duration showed no significant difference.

**Discussion:**

The proposed method demonstrates excellent performance in snoring classification and provides preliminary evidence for exploring acoustic features associated with cerebrovascular stenosis.

## 1 Introduction

### 1.1 Research background

Snoring is a breathing sound generated by the vibration of upper airway tissues that partially collapse during inhalation. It is common, occurring in approximately 50% of adults and 3.2%–12.1% of children (Ersu et al., 2004; Duckitt et al., 2006), and is widely regarded as an early clinical sign of obstructive sleep apnea-hypopnea syndrome (OSAHS) (Jiang et al., 2020), which causes damage to multiple systems throughout the body through intermittent hypoxia and sleep fragmentation. Research has established strong associations between OSAHS and various health conditions, including cardiovascular and cerebrovascular diseases (Zhu et al., 2024; Hong et al., 2022; Raptis et al., 2021), metabolic syndrome (Kargar et al., 2021), and cognitive impairment (Leng et al., 2016; Ylä-Herttuala et al., 2021). Notably, individuals with moderate to severe OSAHS face a stroke risk up to four times higher than that of the general population (Javaheri et al., 2022; Sanchez et al., 2022).

Studies have shown that the prevalence of OSAHS is significantly higher among patients with cerebrovascular diseases, such as stroke, than in the general population (Bassetti et al., 2006). Such patients may simultaneously exhibit upper airway obstruction and central respiratory regulation abnormalities (Tanayapong and Kuna, 2021), and their snoring sounds may present unique acoustic features. Clinical studies have demonstrated a positive correlation between snoring energy in the 652–1,500 Hz frequency band and the common carotid artery intima-media thickness (CCA-IMT) (Lee et al., 2016). Early epidemiological research also suggests a significant association between heavy snoring and carotid atherosclerosis (Lee et al., 2008). Therefore, exploring the acoustic differences between snoring sounds in patients with concomitant cerebrovascular stenosis and those without stenosis may provide new clues about the underlying pathological mechanisms. This highlights the importance of further integrating artificial intelligence (AI) methods into such research.

### 1.2 Related work

In recent years, deep learning (DL) has been extensively applied in snoring detection and recognition, primarily along two directions: multimodal fusion and lightweight portable detection. Regarding multimodal approaches, Li et al. (2025) employed the end-to-end audio classification framework DFNet to fuse patient medical history with physiological information using a multi-branch convolutional architecture, achieving an accuracy of 84.1% in a four-class OSAHS classification task. Qiu et al. (2026) proposed the Multimodal Integration and Missing Audio Reconstruction (MIMAR-OSA) model, which uses multimodal data fusion and missing modality reconstruction strategies to maintain high diagnostic accuracy even when certain signals are unavailable. Gu et al. (Bing, 2025) proposed a hierarchical Transformer model that integrates electroencephalogram (EEG) signals with semantic features of English listening comprehension, significantly enhancing the screening performance for obstructive sleep apnea (OSA) in noisy environments. Hu et al. (2025) proposed the information bottleneck-based parallel CNN-Transformer network (IPCT-Net), which achieves parallel fusion of local and global features, outperforming traditional methods on home sleep test data.

In the realm of lightweight and portable solutions, Zhang R. et al. (2024) developed a lightweight model based on long short-term memory spiking neural networks (LSTM-SNN), optimized through threshold coding and an alternative gradient method, reaching an accuracy of 93.4%. The MinSnore model proposed by Dinh et al. (2025) combines an advanced lightweight network architecture with self-supervised learning pre-training to deliver outstanding performance in practical applications. Sillaparaya et al. (2025) proposed a transfer learning framework that combines MobileNetV3-Large with a modified SENet module, achieving favorable performance on Mel-spectrogram and MFCC inputs under a 10-fold cross-validation, and showing potential for portable sleep apnea monitoring. Hong et al. (2025) achieved high sensitivity and specificity in home settings by combining smartphone recordings with a Vision Transformer model, providing a viable solution for wearable and portable sleep apnea detection.

Although these approaches have contributed substantially to OSAHS diagnosis and snoring classification, two major limitations remain. First, most studies are conducted in controlled laboratory or home environments and lack validation in the complex acoustic environment of real hospital wards. Second, prior work has primarily focused on determining the presence or severity of OSAHS, and few have specifically analyzed patients with concomitant cerebrovascular stenosis as a distinct clinical subgroup.

In this study, we prospectively collected overnight snoring audio from patients diagnosed with OSAHS in a real hospital ward environment. Patients were grouped according to the presence or absence of concomitant cerebrovascular stenosis. The research objectives were as follows: (1) to construct a snoring and non-snoring classification model based on an improved ConvNeXt architecture, (2) to achieve classification between stenotic and non-stenotic snoring, and (3) to explore the acoustic feature differences between these two clinical subgroups and their clinical implications through statistical analysis.

## 2 Materials and methods

### 2.1 The overall framework of the proposed method

**FIGURE 1:**
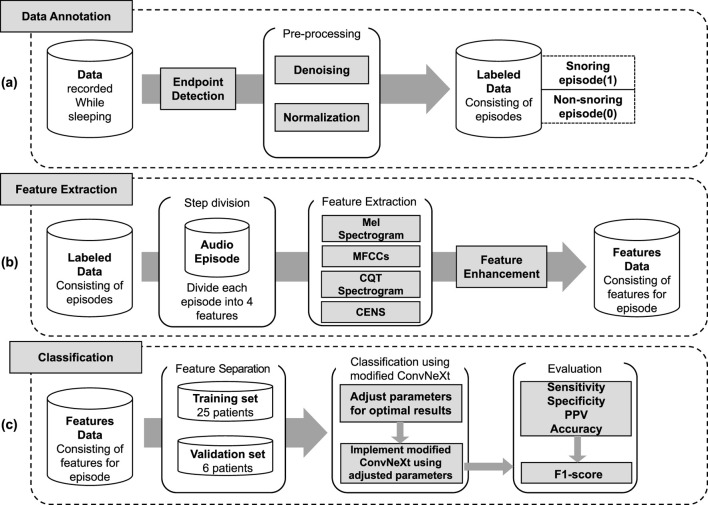
Overall framework of the proposed method. **(a)** Data annotation, including endpoint detection, denoising, normalization, and labeling of snoring (1) and non-snoring (0) episodes. **(b)** Feature extraction, where each labeled audio episode is transformed into four acoustic representations (Mel spectrogram, MFCCs, CQT spectrogram, and CENS), followed by feature enhancement. **(c)** Classification, including data partitioning into training and validation sets, implementation of the modified ConvNeXt model, and evaluation using sensitivity, specificity, positive predictive value (PPV), accuracy, and F1-score.

The proposed snoring classification method, incorporating dynamic convolution and attention mechanisms, consists of three main components: data annotation, feature extraction, and classification. As illustrated in Figure 1, snoring segments are labeled as 1, and non-snoring segments as 0. In the feature extraction phase, labeled audio segments are converted into four types of acoustic features: Mel spectrogram, Mel-frequency cepstral coefficients (MFCCs), Constant Q Transform (CQT) spectrogram, and Chroma Energy Normalized Statistics (CENS). After partitioning the extracted feature dataset into training and validation sets, the data was fed into an enhanced ConvNeXt model to perform the classification task of distinguishing snoring from non-snoring sounds. Furthermore, this framework was extended to classify snoring sounds between patients with cerebrovascular stenosis and those without stenosis, exploring differences in snoring acoustic features across these distinct patient groups.

### 2.2 Data collection

This study collected snoring data from 31 patients at the Traditional Chinese Medicine Department, Zigong First People’s Hospital, using a professional voice recorder and high-sensitivity microphone. Among them, 16 patients had cerebrovascular stenosis (6 mild, 5 moderate, 5 severe), while 15 patients had no stenosis. All patients were diagnosed with OSAHS through polysomnography (PSG). During hospitalization, each patient underwent continuous collection of 8-h nocturnal snoring data covering a full sleep cycle. To ensure data quality and diversity, microphones were positioned 2–3 cm from the patients’ mouths, with signal collection conducted across various hospital environments. All patients underwent clinical evaluation and imaging examinations to confirm the presence or absence of cerebrovascular stenosis, and were subsequently categorized into “stenotic group” and “non-stenotic group” for analysis in follow-up studies.

### 2.3 Data annotation

All raw sleep sound signals were processed using endpoint detection to isolate valid acoustic segments, followed by noise reduction and normalization. Under the guidance of medical professionals, the segments were manually annotated in Audacity. The primary objective of this experiment was to identify snoring segments; thus, the signals were labeled into two categories: “snoring” and “non-snoring.” Snoring segments were annotated based on a complete snoring cycle, ensuring each segment contained a full cycle. Non-snoring segments were selected from periods without snoring events, encompassing various sounds such as breathing, footsteps, coughing, and conversation. To reduce patient-dependent bias in model training and validation, the dataset was organized strictly at the patient level: 25 patients were assigned to the training set and 6 to the validation set. All segments from a given patient were included exclusively in one set, ensuring no segment-level mixing across training and validation. The overall training-to-validation ratio was maintained at 8:2, and this subject-independent strategy was applied consistently to both the “snoring *versus* non-snoring” classification and the “stenotic *versus* non-stenotic” snoring classification tasks. The distribution of audio segments across categories is shown in Table 1.

**TABLE 1:** Dataset distribution.

Dataset	Snoring			Non-snoring			Overall total
	Stenotic	Non-stenotic	Total	Stenotic	Non-stenotic	Total	
Training (25 patients)	16,150	7900	24,050	15,850	9200	25,050	49,100
Validation (6 patients)	4018	1977	5995	4004	2336	6340	12,335
Total (31 patients)	20,168	9877	30,045	19,854	11,536	31,390	61,435

### 2.4 Feature extraction

Previous studies (Hou et al., 2024) have demonstrated that audio spectrograms containing frequency and amplitude information that change over time can distinguish between different types of sounds. Building on this foundation, feature extraction was performed on manually annotated audio segments. Each clip was divided into 20-millisecond (ms) frames using a Hamming window with a 10-m frame shift, and all audio was uniformly resampled to 22.05 kHz using the Python library librosa. After zero-mean normalization, four types of feature maps were generated, with all images standardized to 224 × 224 pixels. Since the snoring event duration in this patient cohort generally lasted less than 3 s, audio was uniformly segmented into 3-s clips before conversion into images. Segments shorter than 3 s were padded with zeros to ensure consistent input length for the model.

#### 2.4.1 Mel spectrogram

The Mel spectrogram extracts time-frequency features by simulating the auditory characteristics of the human ear. First, the audio is pre-emphasis, framing, and windowing. Pre-emphasis is the process of enhancing the high-frequency components of an audio signal using a high-pass filter. The signal is then segmented into short-time frames, and each frame is processed with a Hamming window to reduce spectral discontinuities at the boundaries. Next, the short-time Fourier transform (STFT) is computed for each frame to convert the time-domain signal into a frequency-domain representation (Zhang R. et al., 2024). The formula is shown in Equation 1:

$$X\left(k\right)=\overset{N-1}{\underset{n=0}{\sum }}x\left(n\right)\cdot w\left(n\right)\cdot e^{-j2\pi kn/N},\;k=0,1,\ldots ,N-1$$ (1)

In the formula: x(n) represents the signal value of the n -th sampling point; w(n) is the window function, which is the Hamming window here; N is the number of sampling points in each frame.

Finally, a Mel-scale triangular filter bank that converts linear frequencies to logarithmic frequencies is applied to the power spectrum to obtain the Mel spectrogram. This is achieved using the formula in Equation 2:

$$s\left(m\right)=\ln\left(\overset{N-1}{\underset{k=0}{\sum }}\left|X{\left(k\right)}^{2}H_{m}\left(k\right)\right|\right),0\leq m\leq M$$ (2)

In the formula: H(k) is the frequency response of the triangular filter, m denotes the m -th filter, and M is the number of Mel-scale triangular filter banks.

#### 2.4.2 MFCCs

MFCCs further extract cepstral features based on the Mel spectrogram. After calculating the Mel spectrogram, the first 13 cepstral coefficients are obtained through discrete cosine transform (DCT) (Zhang R. et al., 2024), and the filter coefficients are decoupled. This process separates glottal excitation from vocal tract response, highlighting the static characteristics of the spectrum. The calculation formula is provided in Equation 3:

$$C\left(l\right)=\overset{M}{\underset{m=0}{\sum }}s\left(m\right)\cos\left(\frac{\pi l\left(m-0.5\right)}{M}\right),\;l=1,2,\ldots ,L$$ (3)

Where L represents the dimension of MFCCs, M denotes the number of Mel-scale triangular filter banks, identical to that in Equation 2.

#### 2.4.3 CQT spectrogram

CQT applies a logarithmic frequency scale to enable multi-resolution time-frequency analysis. The audio signal is first preprocessed through steps such as pre-emphasis and normalization. Subsequently, the CQT is computed to generate a time-frequency feature representation, where Q represents the Q factor, i.e., the ratio of the center frequency to the bandwidth (Xie et al., 2021). The Q factor is equal for all frequency intervals. The calculation of the Q factor is based on the two formulas in Equations 4, 5:

$$Q=\frac{f_{k}}{\Delta f_{k}}$$ (4)

$$f_{k}=f_{1}2^{\frac{k-1}{B}}$$ (5)

In the formulas: $\Delta f_{k}$ is the bandwidth; $f_{k}$ is the center frequency of the k -th bin; $f_{1}$ is the center frequency of the lowest frequency bin; B represents the number of bins within each octave frequency.

This method analyzes signals using a set of filters with logarithmic frequency distribution and constant Q values to generate complex spectrograms with high-frequency resolution.

#### 2.4.4 CENS

CENS shows that chroma changes are more robust to temporal and timbre variations (Abraham et al., 2023). To extract CENS features, the audio signal is first processed through pre-emphasis, framing, windowing, and STFT. Then, the chroma information of each frame is extracted through a 12-dimensional chroma filter bank. Finally, temporal smoothing and normalization are performed to generate the CENS feature representation.

### 2.5 Feature enhancement

The critical information in snoring sounds is often concentrated within specific frequency bands or short-term local regions. However, this information can be easily obscured by background noise or irrelevant components in the original feature maps, thereby reducing the model’s ability to detect abnormal patterns. To address this issue, this paper draws on the enhanced spectrogram technique proposed by Wang et al. (2024) and proposes an adaptive filter bank enhancement method. Unlike methods that rely on fixed frequency band settings, the proposed method selects enhancement channels based on energy distribution, making it compatible with all four types of feature maps used in this study. Specifically, it evaluates the energy within each frame and dynamically adjusts filter response weights to emphasize high-energy regions. When the energy output of a filter exceeds the 80th percentile of that frame’s energy distribution, an enhancement factor of β is applied; otherwise, the filter response remains unchanged. The β value was determined via grid search on the validation set of the baseline model in this paper ( β∈{1.1,1.2,…,2.0} , step size 0.1), with β = 1.5 yielding the best performance on the validation set (see Supplementary Table S1). The enhanced filter bank is expressed in Equations 6–8:

$$H_{i}^{\prime }\left(f,t\right)=a_{i}\left(t\right)\cdot H_{i}\left(f\right)$$ (6)

where,

$$a_{i}\left(t\right)=\left\{\begin{array}{c} \beta ,E_{i}\left(P_{t}\right)>percentile_{80}\left(P_{t}\right)\;\\ 1,otherwise\; \end{array}\right.$$ (7)

and

$$E_{i}\left(P_{t}\right)=\underset{f}{\sum }H_{i}\left(f\right)\cdot P_{t}\left(f\right)$$ (8)

In the formulas: $H_{i}\left(f\right)$ represents the response of filter i at frequency f ; $a_{i}\left(t\right)$ represents the enhancement factor of frame t ; β is the enhancement multiple; $\cdot P_{t}\left(f\right)$ represents the power spectrum of audio at frame t and frequency f ; $E_{i}\left(P_{t}\right)$ is the response value of filter i to $P_{t}$ .

**FIGURE 2:**
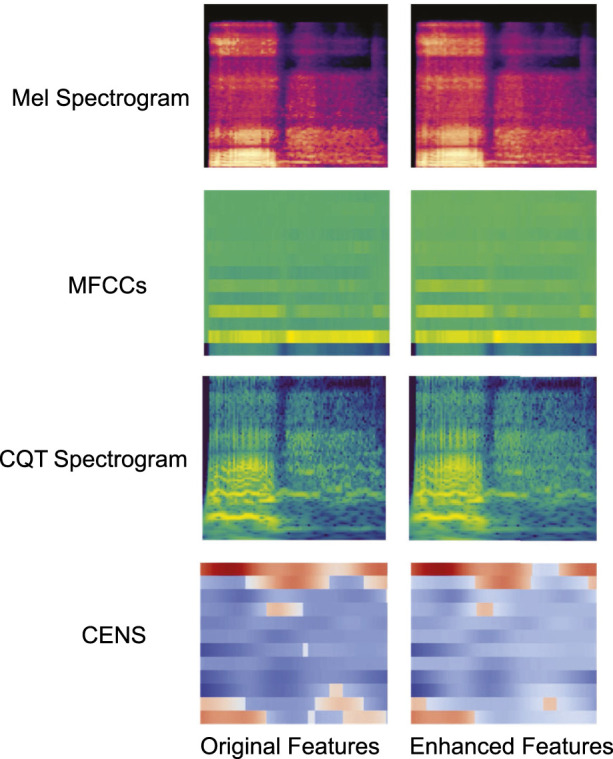
Example of feature map enhancement before and after comparison.

Figure 2 shows examples of visual comparisons of different feature maps before and after enhancement. The original feature maps are shown on the left, and the enhanced feature maps are shown on the right. The high-energy regions in the enhanced feature map are clearer and more prominent.

### 2.6 Classification

**FIGURE 3:**
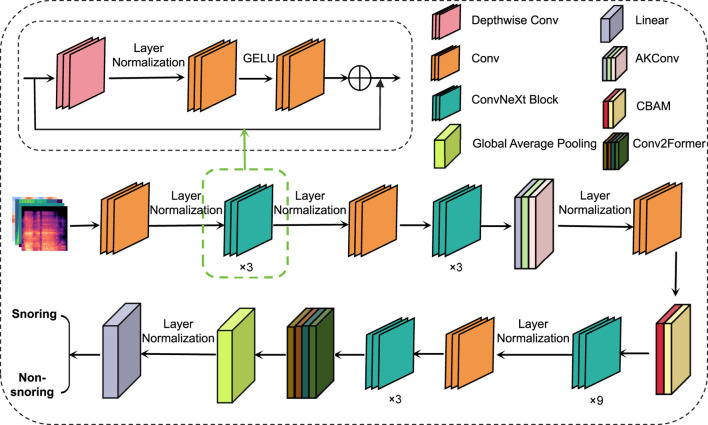
Overall architecture of the model.

This paper proposes a snoring sound classification method that is based on an improved ConvNeXt model integrating dynamic convolution and attention mechanisms. The method consists of four components: the ConvNeXt network, the Alterable Kernel Convolution (AKConv) module, the Convolutional Block Attention Module (CBAM), and the Conv2Former module. ConvNeXt serves as the backbone network, extracting multi-scale acoustic features. The AKConv module enhances adaptability to the irregular spectral features of snoring by dynamically adjusting the convolutional kernel shapes. CBAM utilizes a channel-spatial attention mechanism to emphasize key frequency bands while suppressing background noise. Finally, the Conv2Former module combines local and global feature interactions to effectively model the periodic dependencies of snoring events. This integrated design addresses snoring signals’ characteristics of short-time non-stationarity, cross-band distribution, and periodicity. It enables the model to simultaneously capture both local spatiotemporal features and global periodic structures while enhancing robustness against noise interference, achieving superior feature representation capabilities compared to standalone modules or traditional ConvNeXt architectures. The overall model architecture is illustrated in Figure 3.

#### 2.6.1 ConvNeXt

**FIGURE 4:**
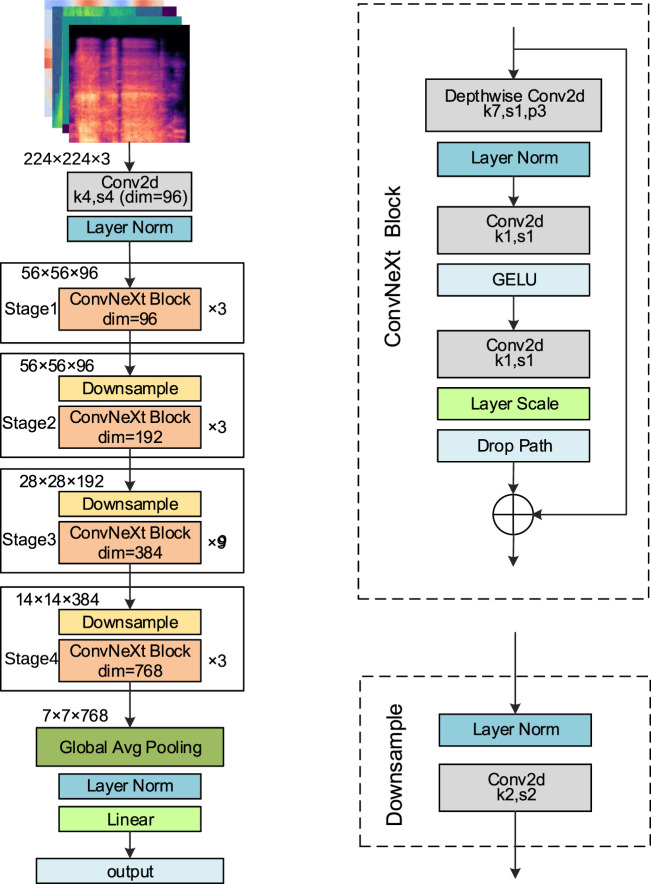
ConvNeXt-T network structure.

ConvNeXt (Liu et al., 2022), developed by Facebook AI Research in 2022, is a high-performance convolutional neural network that integrates key design ideas from Transformer architectures. By introducing large convolutional kernels, streamlining convolutional operations, and optimizing the overall network structure, ConvNeXt achieves substantial gains in both performance and computational efficiency. It is available in four variants: ConvNeXt-Tiny (T), Small (S), Base (B), and Large (L). In this study, the ConvNeXt-T model is adopted as the backbone network. The detailed structure is presented in Figure 4.

The input to the network is a feature map of size 224 × 224 × 3. It first passes through an initial convolutional layer with a 4 × four convolutional kernel and a stride of four, generating a feature map of 56 × 56 × 96, followed by layer normalization (LN) processing. This output then enters Stage 1, where it undergoes feature transformation through three ConvNeXt Blocks, each employing the standard structure of depthwise separable convolutions combined with multilayer perceptron (MLP). After downsampling with a stride of 2, a feature representation with a spatial size of 28 × 28 and 192 channels is obtained, which is then fed into Stage 2 comprising three ConvNeXt blocks. The Stage 2 output is downsampled to 14 × 14 × 384 and input into Stage 3. After another downsampling step, Stage 3 generates a 7 × 7 × 768 feature map, which is then fed into Stage 4. The resulting 7 × 7 × 768 feature map is compressed by global average pooling into a 1 × 1 × 768 vector, followed by LN. Finally, this vector is fed into a linear layer (also called fully connected layer) with an output dimension of 2 (where two represents the number of classification categories), yielding the classification results.

#### 2.6.2 AKConv

**FIGURE 5:**
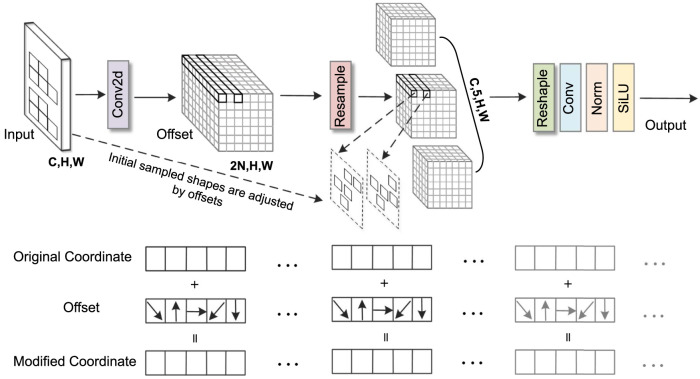
AKConv network structure.

In snoring sound classification tasks, traditional convolution operations struggle to effectively capture irregular snoring feature patterns in spectrograms. To address this, this study introduces the AKConv module (Zhang et al., 2023) into Stage 2 of ConvNeXt. Its core advantage lies in dynamically adjusting the shape and number of parameters of convolutional kernels to adaptively match the diverse morphological features of the snoring spectrum. In this study, the number of core parameters (num_param) was set to three to balance model complexity and representational capacity, while the convolution stride was fixed at one to preserve spatial resolution. As illustrated in Figure 5, the AKConv module takes a spectrogram with dimensions (C, H, W) as input. It first determines the initial sampling position of the convolutional kernel through a coordinate generation algorithm. Specifically, the origin is fixed at the upper-left corner (0,0) as the common reference point for all kernel samples. The grid range is obtained by dynamically computing a base size (base_int) and handling the remainder: a regular grid region is first constructed, then the remaining points are added, resulting in an initial sampling layout that adapts to any number of parameters. Subsequently, a Conv2d layer is employed to learn spatial offsets of shape (B, 2N, H, W). The weights of this offset prediction layer are initialized to zero and undergo learning rate decay of 0.1 during backpropagation to ensure training stability. Subsequently, feature resampling is performed on the adjusted sampling points to precisely capture the deformed feature regions. Finally, the resampled features undergo dimension reshaping, convolution operations, and normalization processing. The optimized feature representations are then output via the SiLU activation function. It is worth noting that AKConv is an early variant of dynamic convolution. Its successor, LDConv (Zhang X. et al., 2024), further improves computational efficiency. However, AKConv was used in all experiments conducted in this paper.

#### 2.6.3 CBAM

**FIGURE 6:**
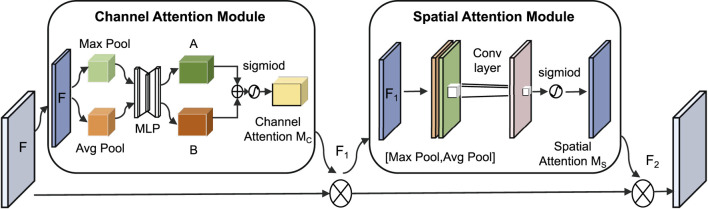
CBAM network structure.

In this classification task, key frequency bands in the spectrogram (such as snoring harmonics) are often intermingled with background noise in both the spatial and channel dimensions. To address this specificity, this paper introduces the CBAM (Woo et al., 2018), which combines the Channel Attention Module (CAM) and the Spatial Attention Module (SAM). Its core function is to highlight important features through dual-path weighting, as shown in Figure 6.

The CAM performs max pooling and average pooling on the input feature map F (output size 1 × 1), respectively, passing through a shared two-layer MLP (with a reduction ratio of 16) to obtain feature maps A and B. The sum of these two feature maps is passed through a sigmoid function to generate the channel attention weight $M_{C}\left(F\right)$ , which enhances key frequency bands associated with snoring sounds. The calculation formulas are given in Equations 9, 10:

$$M_{C}\left(F\right)=\sigma \left(W_{1}\left(W_{0}\left(F_{\text{avg}}^{C}\right)\right)+W_{1}\left(W_{0}\left(F_{\text{max}}^{C}\right)\right)\right)$$ (9)

$$F_{1}=M_{C}\left(F\right)⊙F$$ (10)

In the formulas: $M_{C}\left(F\right)$ is the output weight of channel attention; σ is the sigmoid activation function; $W_{0}$ and $W_{1}$ are the weights of the first and second fully connected layers, respectively; $F_{\mathrm{avg}}^{C}$ and $F_{\max}^{C}$ represent average pooling and max pooling at the channel level, respectively; $F_{1}$ denotes the channel attention feature map obtained by weighting the original feature map F ; and ⊙ denotes element-wise multiplication.

The SAM performs max pooling and average pooling on the feature map $F_{1}$ , then concatenates and fuses the results. It undergoes a 7 × 7 convolution with 3-stride padding to reduce the dimension to a single channel. Following this, a sigmoid function generates the spatial attention weights $M_{S}\left(F_{1}\right)$ , focusing on regions with concentrated energy in the spectrum. The calculation formulas are presented in Equations 11, 12:

$$M_{S}\left(F_{1}\right)=\sigma \left(f^{7\times 7}\left(\left[F_{\text{avg}}^{S};F_{\text{max}}^{S}\right]\right)\right)$$ (11)

$$F_{2}=M_{S}\left(F_{1}\right)⊙F_{1}$$ (12)

In the formulas: $M_{S}\left(F_{1}\right)$ is the output weight of spatial attention; $f^{7\times 7}$ is a 7 × 7 convolution kernel; $F_{\mathrm{avg}}^{S}$ and $F_{\max}^{S}$ are average pooling and max pooling at the spatial level, respectively; $F_{2}$ denotes the final output feature map of the CBAM module after spatial attention; and ⊙ denotes element-wise multiplication.

#### 2.6.4 Conv2Former

**FIGURE 7:**
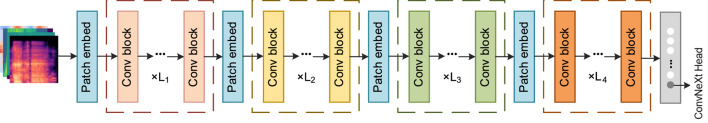
Conv2Former network structure.

For the modeling requirements of long-range dependencies in snoring signals, traditional self-attention mechanisms are often employed. However, these methods typically incur high computational costs, which limit their practical application. To solve this problem, this paper introduces the Conv2Former module (Hou et al., 2024) in Stage 4, whose structure is shown in Figure 7. This module is divided into four stages, each with different image resolutions, channel counts, and numbers of convolutional modulation blocks (Conv blocks). Adjacent stages reduce image resolution while increasing the number of channels through a patch embedding block (patch embed), employing a pyramid-like structure to extract features of different granularities. The Conv block replaces the self-attention layer in the Transformer with a convolutional modulation (ConvMod) layer. The key parameters of the ConvMod used in this study are as follows: the input and output dimensions are set to 768, matching the channel number of Stage 4; internally, an 11 × 11 depthwise separable convolution with padding of 5 is applied to preserve resolution, allowing the large convolutional kernel to encode spatial features and enhance global information modeling.

### 2.7 Statistical methods and 5-fold cross-validation

Statistical analyses were performed using SPSS version 26.0. Continuous variables were first tested for normality. Normally distributed data are expressed as mean ± standard deviation (SD) and compared between groups using the independent-samples t-test, whereas non-normally distributed data are expressed as median (interquartile range, IQR) and compared using the Mann-Whitney U test. Categorical variables were presented as counts and percentages, and group comparisons were conducted using the Chi-square $\left(\chi^{2}\right)$ test or Fisher’s exact test when appropriate.

In ablation experiments, models were evaluated using 5-fold cross-validation based on patient grouping. Data from 31 patients were divided into five folds at the patient level, with each fold containing complete data from distinct patients (detailed statistics are provided in Supplementary Table S2). This ensured strict subject independence between folds and guaranteed that no segment-level information from the same patient appeared in both training and validation sets. In each iteration, one fold was used for validation and the remaining four for training. After repeating this process five times, each fold had been used as the validation set once. Results are presented as mean ± SD for each fold. Differences between the model in this study and models with added modules were compared using the Wilcoxon signed-rank test. The significance level was set at p<0.05 , with significant differences marked with “ ∗ ” in the tables.

## 3 Results

### 3.1 Experiment setup

This experiment was conducted in the following software and hardware environment: the processor is an Intel® Xeon® Silver 4210R (2.40 GHz), equipped with an NVIDIA RTX 3080 (10 GB) graphics card, and the operating system is Ubuntu 20.04.6. The deep learning framework used is PyTorch 2.4.1 (CUDA 11.8), running in a Python 3.8.20 environment. During training, the batch size was set to 32, the AdamW optimizer was used for parameter updates, the initial learning rate was $2\times 10^{-4}$ , and the weight decay coefficient was 0.05. The model was trained for 100 epochs, using the cross-entropy loss function to supervise the optimization process of the binary classification task.

### 3.2 Evaluation criteria

This paper uses five metrics to evaluate model performance, including accuracy, sensitivity, specificity, positive predictive value (PPV), and F1-score. Accuracy represents the overall proportion of correctly classified samples and serves as a basic indicator of classification performance. Sensitivity measures the model’s ability to correctly identify actual snoring events, while specificity reflects its capacity to correctly recognize non-snoring samples. PPV refers to the proportion of true snoring samples among those predicted as snoring. F1-score provides a comprehensive assessment of the model’s robustness under imbalanced category conditions.

The calculation formulas are shown in Equations 13–17:

$$Accuracy=\frac{TP+TN}{TP+TN+FP+FN}$$ (13)

$$\text{Sensitivity}=\frac{TP}{TP+FN}$$ (14)

$$\text{Specificity}=\frac{TN}{TN+FP}$$ (15)

$$PPV=\frac{TP}{TP+FP}$$ (16)

$$F1-score=\frac{2PPV•\text{Sensitivity}}{PPV+\text{Sensitivity}}$$ (17)

Where TP , TN , FP , and FN represent true positive, true negative, false positive, and false negative, respectively.

### 3.3 Performance evaluation of feature enhancement

To verify the applicability and effectiveness of the feature enhancement method proposed in this paper on different feature maps, a comparison experiment was conducted between the Mel spectrogram and CQT spectrogram before and after enhancement using the method proposed in this paper. The comparison results are shown in Table 2. The experimental results show that the two enhanced spectrograms are superior to the unenhanced ones in all evaluation metrics, indicating that the adaptive filter bank enhancement method proposed in this paper can effectively improve the representation ability of key information in the feature map.

**TABLE 2:** Comparison of results before and after feature enhancement (Unit: %).

Feature map	Accuracy	Sensitivity	Specificity	PPV	F1-score
Mel Spectrogram	89.10	88.55	89.73	89.05	89.08
Enhanced Mel Spectrogram	90.24	89.67	90.88	90.25	90.24
CQT Spectrogram	87.46	87.37	87.67	87.59	87.51
Enhanced CQT Spectrogram	88.35	88.32	88.72	88.35	88.37

PPV, positive predictive value.

### 3.4 Comparative experiment

#### 3.4.1 Snoring *versus* non-snoring classification

To validate the effectiveness of the proposed method, we conducted comparative experiments between snoring and non-snoring sounds using the four enhanced acoustic feature maps against the following eight classical classification networks. The experimental results are shown in Table 3.

**TABLE 3:** Comparison of classification performance for snoring and non-snoring sounds (Unit: %).

Feature map	Model	Accuracy (95% CI)	Sensitivity	Specificity	PPV	F1-score
Mel Spectrogram	ViT-B (Dosovitskiy et al., 2021)	76.43 (65.37–82.91)	77.36	78.21	78.52	78.03
	MobileNetV3-L (Sillaparaya et al., 2025)	82.51 (73.23–89.57)	81.88	83.02	82.24	82.37
	DFNet (Li et al., 2025)	84.32 (77.11–90.07)	83.57	84.80	84.15	84.16
	Swin-T (Liu et al., 2021)	84.26 (76.48–90.81)	83.31	85.17	84.39	84.60
	XCiT-T (El-Nouby et al., 2021)	83.09 (74.02–89.96)	83.13	83.76	83.21	83.20
	ResNet50 (He et al., 2016)	85.87 (79.36–91.79)	84.79	86.78	86.73	85.85
	DenseNet121 (Huang et al., 2017)	86.45 (80.47–91.63)	82.37	86.69	86.25	86.11
	ConvNeXt-T (Liu et al., 2022)	88.16 (82.97–92.54)	87.85	88.24	88.19	88.07
	This paper	90.24 (85.31–94.67)	89.67	90.88	90.25	90.24
MFCCs	ViT-B (Dosovitskiy et al., 2021)	69.45 (58.92–78.33)	68.76	69.88	70.12	69.50
	MobileNetV3-L (Sillaparaya et al., 2025)	79.00 (70.12–86.41)	78.31	79.52	79.03	78.87
	DFNet (Li et al., 2025)	81.31 (74.05–87.29)	80.85	81.66	81.25	81.30
	Swin-T (Liu et al., 2021)	82.31 (75.19–88.77)	82.77	81.73	82.27	82.34
	XCiT-T (El-Nouby et al., 2021)	79.58 (71.47–86.97)	79.67	78.81	79.79	79.82
	ResNet50 (He et al., 2016)	83.57 (77.41–89.21)	83.50	83.32	83.61	83.62
	DenseNet121 (Huang et al., 2017)	84.15 (78.33–89.62)	84.57	84.06	84.25	84.27
	ConvNeXt-T (Liu et al., 2022)	86.84 (81.40–91.27)	87.49	86.32	86.89	86.89
	This paper	89.11 (83.77–93.52)	89.44	88.67	89.12	89.21
CQT Spectrogram	ViT-B (Dosovitskiy et al., 2021)	77.38 (67.90–85.73)	76.82	78.43	78.25	77.64
	MobileNetV3-L (Sillaparaya et al., 2025)	80.55 (72.30–87.92)	79.68	81.23	80.46	79.94
	DFNet (Li et al., 2025)	82.03 (75.12–87.86)	81.55	82.67	82.14	82.00
	Swin-T (Liu et al., 2021)	83.05 (76.05–89.40)	82.14	84.11	83.30	83.22
	XCiT-T (El-Nouby et al., 2021)	82.33 (74.50–88.98)	82.42	82.69	82.37	82.38
	ResNet50 (He et al., 2016)	84.05 (78.10–89.65)	83.97	84.81	84.52	84.12
	DenseNet121 (Huang et al., 2017)	84.52 (79.12–89.48)	84.70	84.38	84.52	84.53
	ConvNeXt-T (Liu et al., 2022)	86.14 (81.02–90.76)	85.61	86.65	86.13	86.15
	This paper	88.35 (83.77–92.80)	88.32	88.72	88.35	88.37
CENS	ViT-B (Dosovitskiy et al., 2021)	66.89 (55.97–76.52)	74.63	59.14	64.57	69.23
	MobileNetV3-L (Sillaparaya et al., 2025)	73.59 (63.84–81.97)	74.00	72.80	73.12	73.25
	DFNet (Li et al., 2025)	76.16 (68.10–83.94)	76.30	75.52	76.07	76.13
	Swin-T (Liu et al., 2021)	77.20 (69.02–83.98)	79.43	75.11	77.24	77.25
	XCiT-T (El-Nouby et al., 2021)	77.15 (69.05–83.84)	76.67	76.70	76.71	76.68
	ResNet50 (He et al., 2016)	82.87 (77.10–88.24)	82.24	83.55	83.78	82.91
	DenseNet121 (Huang et al., 2017)	81.66 (75.98–87.32)	80.62	82.45	82.59	81.63
	ConvNeXt-T (Liu et al., 2022)	84.37 (79.01–89.40)	84.19	84.58	84.44	84.40
	This paper	87.94 (82.83–92.45)	87.05	88.94	87.88	87.96

The MobileNetV3-L results in this table refer only to the backbone, without the modified SENet, module used in (Sillaparaya et al., 2025). In this study, only the backbone was reproduced to ensure consistency in the comparative experiments. CI: confidence interval.

For statistical robustness, classification accuracy was further reported with 95% confidence intervals (CIs) estimated by patient-level bootstrap (B = 1,000 resamples, seed = 42), where each patient was treated as the sampling unit to avoid segment-level leakage. As shown in Table 3, the proposed method significantly outperformed the comparison models in all evaluation metrics for the four acoustic feature maps. Among them, the Mel spectrogram achieved the highest classification accuracy at 90.24% (95% CI: 85.31%–94.67%), followed by MFCCs, CQT spectrogram, and CENS. The overall performance of the lightweight models MobileNetV3 and DFNet was lower than that of medium-sized models such as ResNet50 and DenseNet121. The ConvNeXt baseline model outperformed the traditional CNN architectures DenseNet121 and ResNet50, with accuracy gains of 1.71% and 2.29%, respectively, on the Mel spectrogram. Compared to the ConvNeXt baseline model, our method achieved approximately 2% improvements in accuracy, sensitivity, specificity, PPV, and F1-score on the Mel spectrogram. Accuracy gains on MFCCs, CQT spectrogram, and CENS also ranged from 2% to 4%. Overall, our method maintained a consistent advantage across models of different scales, and its F1-score and accuracy remained closely aligned, supporting its robustness. We also evaluated performance at the patient level through majority voting across segments (Supplementary Table S3). In the validation set (n = 6 patients), the proposed model correctly classified all patients (100.00% accuracy), whereas the ConvNeXt baseline reached 83.33% (5/6). This indicates that the improvement observed at the segment level is preserved at the clinically relevant per-patient level.

**FIGURE 8:**
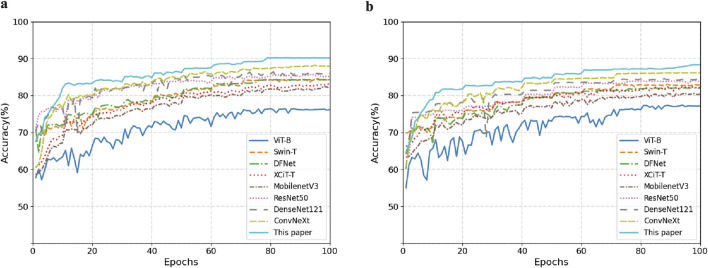
Comparison of validation set accuracy across different feature representations. **(a)** Mel Spectrogram. **(b)** CQT Spectrogram.

Figure 8 shows the comparison of model accuracy between the Mel spectrogram and CQT spectrogram on the validation set. With the exception of the Vision Transformer network, which exhibited performance instability, all other models achieved accuracy above 80%. This paper’s method demonstrates optimal convergence stability on different feature maps. In summary, the effective fusion of dynamic convolution and attention mechanisms enhances the model’s ability to discriminate the time-frequency features of snoring.

#### 3.4.2 Stenotic *versus* non-stenotic snoring classification

To investigate the characteristic differences between snoring sounds from patients with cerebrovascular stenosis and those without stenosis, we selected one representative model from each scale (small, medium, and large) for classification experiments and comparisons. Similarly, accuracy was reported with 95% CIs estimated using patient-level bootstrap (B = 1,000 resamples, seed = 42) to ensure subject independence. As summarized in Table 4, all metric values of the proposed model outperformed other baseline models, with an accuracy of 84.68% (95% CI: 76.03%–89.62%) on the Mel spectrogram. These findings indicate that the proposed method demonstrates potential in distinguishing between stenotic and non-stenotic snoring sounds; however, these results should be regarded as preliminary due to the limited cohort size.

**TABLE 4:** Comparison of classification performance for stenotic and non-stenotic snoring sounds (Unit: %).

Feature map	Model	Accuracy (95% CI)	Sensitivity	Specificity	PPV	F1-score
Mel Spectrogram	ViT-B	69.58 (57.12–79.24)	69.30	69.92	69.55	69.35
	MobileNetV3-L	77.34 (66.20–85.92)	77.65	77.05	77.21	77.43
	ResNet50	81.92 (73.05–88.48)	81.40	82.18	81.85	81.70
	ConvNeXt-T	82.41 (74.22–88.95)	81.76	83.05	82.49	82.28
	This paper	84.68 (76.03–89.62)	84.12	84.35	84.92	84.97

To further validate the robustness of these findings at a clinically relevant scale, we conducted patient-level analysis, as summarized in Supplementary Table S4. When predictions were aggregated per patient in the validation set (n = 6), the proposed model achieved 83.33% accuracy (5/6 patients correctly classified) and identified all stenotic patients with 100.00% sensitivity (4/4), compared with 66.67% accuracy (4/6) for the ConvNeXt baseline. This suggests that the method may prioritize stenotic patients with high sensitivity, even with a small cohort.

### 3.5 Ablation experiment

To evaluate the impact of each proposed module on the performance of the ConvNeXt baseline, ablation experiments were conducted by incorporating different modules onto the four enhanced feature maps for snoring *versus* non-snoring classification. The results are summarized in Table 5. Performance varies by module and feature map. On the Mel spectrogram, the AKConv module demonstrates the most significant performance, achieving an accuracy of 89.62%, which represents a 1.46 percentage point improvement over the baseline ConvNeXt model, while also increasing specificity to 90.05%. This suggests that dynamically adjusting the shape of convolutional kernels enhances adaptability to irregular snoring spectra. Similarly, the AKConv module performs best on the MFCCs, improving specificity by 1.9%. The CBAM module excels on the CQT spectrogram, increasing sensitivity by 2.04%. The Conv2Former module contributes most significantly to the CENS map, improving accuracy by 1.97% and demonstrating its strength in global feature modeling.

**TABLE 5:** Ablation experiment results (Unit: %).

Feature map	Model	Accuracy	Sensitivity	Specificity	PPV	F1-score
Mel Spectrogram	ConvNeXt-T	88.16	87.85	88.24	88.19	88.07
	AKConv	89.62	89.10	90.05	89.58	89.62
	CBAM	89.11	88.65	89.45	89.06	89.08
	Conv2Former	88.75	88.40	88.98	88.80	88.77
	This paper	90.24	89.67	90.88	90.25	90.24
MFCCs	ConvNeXt-T	86.84	87.49	86.32	86.89	86.89
	AKConv	88.45	88.83	88.22	88.41	88.46
	CBAM	88.14	88.40	87.85	88.05	88.12
	Conv2Former	87.95	88.12	87.72	87.90	87.96
	This paper	89.11	89.44	88.67	89.12	89.21
CQT Spectrogram	ConvNeXt-T	86.14	85.61	86.65	86.13	86.15
	AKConv	87.10	86.95	87.24	87.06	87.00
	CBAM	87.92	87.65	88.20	87.90	87.88
	Conv2Former	87.35	87.40	87.28	87.34	87.33
	This paper	88.35	88.32	88.72	88.35	88.37
CENS	ConvNeXt-T	84.37	84.19	84.58	84.44	84.40
	AKConv	85.18	84.68	85.52	85.08	85.14
	CBAM	85.75	85.23	86.25	85.74	85.72
	Conv2Former	86.34	86.02	86.23	86.33	86.34
	This paper	87.94	87.05	88.94	87.88	87.96

**FIGURE 9:**
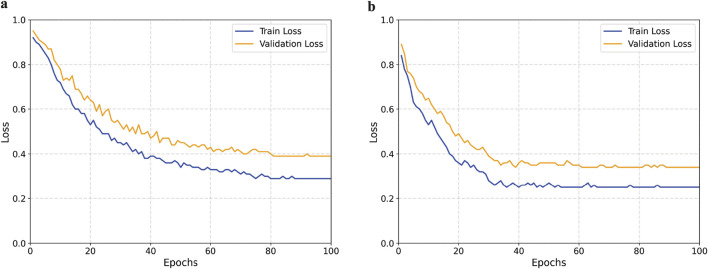
Comparison of training and validation loss curves between the baseline and proposed models. **(a)** Baseline model. **(b)** Proposed model.

To further validate the effectiveness of the proposed method, we plotted the loss curves of the baseline model and our model on both the training and validation sets. As shown in Figure 9, our method converges faster during training and achieves a lower final loss value, indicating that the introduced module enhances feature extraction capabilities and improves the model’s classification performance.

We further validated module performance using 5-fold cross-validation on the Mel spectrogram and assessed significance with the Wilcoxon test (Table 6). The ConvNeXt-T model achieved an average accuracy of 88.24%. Introducing different modules on this foundation yielded performance improvements, with AKConv demonstrating the most prominent enhancement. CBAM and Conv2Former also delivered gains, but the differences compared to our proposed model were statistically significant (p<0.05) . This indicates that while each module contributes to feature enhancement, our model achieves the best overall performance.

**TABLE 6:** Comparison of 5-fold cross-validation performance for ablation experiments on the Mel spectrogram (Unit: %).

Model variant	Accuracy	Sensitivity	Specificity	PPV	F1-score
ConvNeXt-T	88.24 ± 0.88^*^	87.91 ± 0.95^*^	88.32 ± 0.90^*^	88.27 ± 0.93^*^	88.19 ± 0.91^*^
+AKConv	89.57 ± 0.93^*^	89.12 ± 1.01^*^	90.03 ± 0.88^*^	89.51 ± 1.06^*^	89.59 ± 0.92^*^
+CBAM	89.18 ± 0.95^*^	88.69 ± 1.03^*^	89.56 ± 0.91^*^	88.87 ± 1.08^*^	89.11 ± 0.94^*^
+Conv2Former	88.76 ± 0.97^*^	88.33 ± 1.05^*^	88.01 ± 0.93^*^	88.42 ± 1.11^*^	88.78 ± 0.96^*^
This paper	90.31 ± 0.82	89.74 ± 0.95	90.88 ± 0.91	90.28 ± 0.97	90.34 ± 0.89

Results are reported as mean ± SD over 5-fold cross-validation. Values marked with ^*^ indicate significant differences compared with the proposed model, based on the Wilcoxon signed-rank test (p<0.05) .

### 3.6 Computational efficiency comparison analysis

To evaluate the computational efficiency, several representative models were compared on the dataset in this paper, as shown in Table 7. It can be observed that ResNet50 achieves the lowest latency of 12.05 m and the highest throughput of 82.98 images per second (img/s) on the GPU, but its latency increases significantly during CPU inference. MobileNetV3, as a lightweight model with minimal parameters and floating-point operations per second (FLOPs), performs relatively fast on the GPU but underperforms compared to ConvNeXt-T on the CPU. Although Swin-T has a large number of parameters, it exhibits the highest GPU latency and lowest throughput. ConvNeXt-T demonstrates a good balance on both GPU and CPU, with relatively stable latency and throughput. In comparison, the proposed model in this paper increases both parameters and computational cost, resulting in slightly lower inference latency and throughput than ConvNeXt-T. Nevertheless, it still significantly outperforms Swin-T and other lightweight models. It achieves the highest recognition accuracy while maintaining good computational efficiency, offering the best overall performance.

**TABLE 7:** Computational efficiency comparison of different models.

Model	Params (M)	FLOPs (G)	GPU Latency (ms)	GPU Throughput (Img/s)	CPU Latency (ms)	CPU Throughput (Img/s)
ResNet50	23.51	4.13	12.05	82.98	4043	0.25
MobileNetV3	4.20	0.23	13.08	76.40	2900	0.34
Swin-T	27.52	3.13	39.41	25.37	2139	0.47
ConvNeXt-T	27.82	4.49	15.03	65.20	2200	0.45
This paper	32.05	5.20	16.12	61.80	2398	0.42

Params: number of parameters. FLOPs: floating-point operations per second. img/s: images processed per second.

### 3.7 Noise robustness experiment

To evaluate the generalization ability of the proposed method in real-world environments, noise robustness experiments were conducted. Background noise was selected from the publicly available MUSAN dataset (Snyder et al., 2015), which contains speech, music, and various environmental sounds, to simulate interference in hospital and daily settings. For the snoring *versus* non-snoring classification validation set, each audio segment was linearly mixed with MUSAN noise at different signal-to-noise ratios (SNR = +20, +10, 0, −5 dB), with noise energy scaled to achieve the target SNR. The mixed audio segments were preprocessed following the same procedures as in the model training phase and converted into Mel spectrograms for model input.

Table 8 summarizes the performance of our method and the ConvNeXt-T baseline model under different noise conditions. Under mild noise (+20 dB), both models exhibit only a slight decline compared to the noise-free condition. As the noise level increases, our method shows a significantly slower performance degradation, reflecting its stronger resistance to interference. At 0 dB SNR, our model maintains an F1-score of 83.36%, significantly outperforming the baseline model. Even under extreme noise conditions ( −5 dB), it still achieves an F1-score of 78.62%, exceeding the baseline model by over 8 percentage points. These results conclusively demonstrate that the introduced module enhances the model’s feature extraction and discrimination capabilities in complex acoustic environments, thereby improving its robustness and practicality in real-world scenarios.

**TABLE 8:** Performance comparison of proposed model and ConvNeXt-T under different SNR conditions.

Model	SNR (dB)	Performance (%)				
		Accuracy	Sensitivity	Specificity	PPV	F1-score
This paper	∞	90.24	89.67	90.88	90.25	90.24
	+20	89.12	88.75	89.53	88.87	88.82
	+10	86.65	85.20	87.25	85.59	85.87
	0	83.58	82.11	83.79	83.26	83.36
	−5	78.32	77.05	78.16	77.97	78.62
ConvNeXt-T	∞	88.16	87.75	88.24	88.19	88.07
	+20	86.20	85.89	86.95	86.13	85.94
	+10	82.67	81.52	82.89	81.92	82.05
	0	77.58	76.33	78.05	77.43	77.14
	−5	70.96	69.74	71.35	70.30	70.58

SNR: signal-to-noise ratio.

### 3.8 Visualization experiment

**FIGURE 10:**
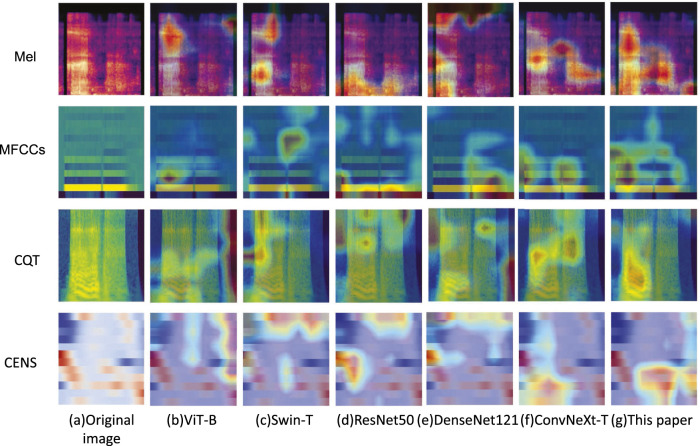
Comparative Grad-CAM visualizations of feature attention among different models.

In order to assess the feature extraction capability of the proposed model, Grad-CAM (Selvaraju et al., 2017) was employed to visualize the feature maps from the final layer of the feature extraction network. Four types of acoustic representations from a randomly selected snoring segment were used as input. Then, ViT-B, Swin-T, ResNet50, DenseNet121, and ConvNeXt networks were chosen for feature map visualization experiments. The results are shown in Figure 10. Taking the Mel spectrogram and CQT spectrogram as examples, our method demonstrates greater focus on the key frequency bands of snoring compared to the other five networks, whose focus appears more dispersed. This confirms that our approach captures more discriminative acoustic features.

### 3.9 Comparative analysis of stenotic and non-stenotic patients

#### 3.9.1 Demographic characteristics

Beyond algorithmic research, we also conducted an in-depth analysis of the clinical characteristics of the study subjects. Table 9 summarizes the baseline demographic characteristics and comorbidities of patients in the stenotic and non-stenotic groups. No significant differences were observed between the two groups in terms of age, gender distribution, hypertension, diabetes, atherosclerosis, dyslipidemia, or apnea-hypopnea index (AHI) (p>0.05) . Given that cerebral infarction and cerebral ischemia are common clinical consequences of cerebrovascular stenosis, only descriptive statistics were performed for these two medical histories. Overall, the two groups were comparable in primary demographic and vascular risk factors, providing a reliable foundation for analyzing snoring-related characteristics.

**TABLE 9:** Baseline characteristics of patients in stenotic and non-stenotic groups.

Item	Stenotic group (n = 16)	Non-stenotic group (n = 15)	$t/\chi^{2}$	p- ${\text{value}}^{a}$
Age (years)	71.44 ± 7.70	67.53 ± 9.12	1.284	0.210
Sex			0.819	0.366
Female	7 (43.8)	9 (60.0)		
Male	9 (56.2)	6 (40.0)		
Hypertension	11 (68.8)	9 (60.0)	0.259	0.611
Diabetes	8 (50.0)	6 (40.0)	0.313	0.576
Atherosclerosis	7 (43.8)	6 (40.0)	0.045	0.833
Dyslipidemia	6 (37.5)	5 (33.3)	0.000	1.000
AHI (events/h)	16.05 ± 5.90	13.75 ± 3.80	1.283	0.210
Cerebral infarction	4 (25.0)	0 (0.0)	—	—
Cerebral ischemia	3 (18.8)	0 (0.0)	—	—

Data are expressed as mean ± SD or number (%). ^*a*^Continuous variables were compared using independent-samples *t*-test, and categorical variables were compared using the $\chi^{2}$ test, as appropriate. AHI: apnea-hypopnea index.

#### 3.9.2 Analysis of differences in snoring acoustic features

We used the Mann-Whitney U test to analyze differences between the stenotic group and the non-stenotic group in three aspects: low-frequency energy ratio (<650 Hz energy proportion), snoring frequency, and snoring event duration. Snoring event duration was calculated by statistically determining the start and end points of each independent snoring event and measuring its duration; the median duration of all events per patient was used as the representative value. The low-frequency energy ratio was calculated based on the power spectral density (PSD) of each snoring segment. Specifically, the ratio of energy in the <650 Hz band to total energy was used as the low-frequency energy ratio for that segment. The median value across all segments for each patient was then taken as the individual indicator. The 650 Hz threshold referenced the 652 Hz threshold proposed by Lee et al. (Lee et al., 2016) and was rounded for computational simplicity.

Results shown in Table 10 indicate that the “low-frequency energy ratio” was significantly lower in the stenotic group than in the non-stenotic group (p = 0.025). This suggests that snoring energy distribution in stenotic patients tends toward high-frequency components, consistent with previous findings showing a positive correlation between snoring energy in the 652–1,500 Hz band and CCA-IMT (Lee et al., 2016). Regarding snoring frequency, the stenotic group exhibited significantly more nocturnal snoring events per 8-h sleep period compared to the non-stenotic group (p = 0.031). This likely reflects more frequent upper airway obstruction or turbulence, aligning with Lee et al.’s (Lee et al., 2008) conclusion that “heavy snoring is significantly associated with carotid atherosclerosis.” In contrast, snoring event duration was slightly longer in the stenotic group but did not reach statistical significance (p = 0.185), likely due to the limited sample size or substantial individual variability. In summary, while this study did not identify significant intergroup differences in snoring event duration, the variations in low-frequency energy ratio and snoring frequency provide preliminary acoustic evidence for assessing cerebrovascular stenosis risk through snoring acoustic features, offering potential clinical implications. Future research should incorporate larger samples and quantitative respiratory parameters from PSG for in-depth validation.

**TABLE 10:** Comparison of snoring characteristics between stenotic and non-stenotic groups.

Group	Low-frequency energy ratio	Snoring frequency (events/8 h)	Snoring event duration (s)
Stenosis group (n = 16)	0.52 (0.46–0.59)	835.50 (667.50–1,341.50)	1.60 (1.48–1.82)
Non-Stenosis group (n = 15)	0.69 (0.58–0.77)	649.00 (434.00–764.50)	1.51 (1.37–1.65)
Z	−2.253	2.174	1.344
p-value	0.025	0.031	0.185

Data are expressed as median (IQR). p values are reported to three decimal places. Statistical comparisons were performed using the Mann-Whitney U test. Significance level: p<0.05 .

To control for potential confounding effects of a history of cerebral infarction or cerebral ischemia, a sensitivity analysis was conducted after excluding these patients. As shown in Table 11, the Mann-Whitney U test revealed that the low-frequency energy ratio in the stenotic group remained significantly lower than that in the non-stenotic group (p = 0.038), and the snoring frequency was significantly higher (p = 0.018); while the difference in snoring event duration between the two groups remained statistically insignificant (p = 0.294). These findings indicate that even after controlling for the confounding effects of cerebral complications, the characteristics of high-frequency shift in snoring energy distribution and increased snoring frequency in stenotic patients persist stably, further validating the reliability of the aforementioned analysis.

**TABLE 11:** Sensitivity analysis of snoring characteristics after excluding cerebral infarction/ischemia cases.

Group	Low-frequency energy ratio	Snoring frequency (events/8 h)	Snoring event duration (s)
Stenosis group (n = 12)	0.52 (0.46–0.57)	835.50 (697.75–1,341.50)	1.59 (1.48–1.82)
Non-Stenosis group (n = 15)	0.69 (0.58–0.77)	649.00 (434.00–764.50)	1.51 (1.37–1.65)
Z	−2.098	2.390	1.073
p-value	0.038	0.018	0.294

Data are expressed as median (IQR). p values are reported to three decimal places. Statistical comparisons were performed using the Mann-Whitney U test. Significance level: p<0.05 .

## 4 Discussion

The ConvNeXt model proposed in this study, which integrates dynamic convolution and attention mechanisms, outperformed baseline networks in snoring sound classification and maintained stable performance under noisy conditions. Statistical analysis revealed significant differences in specific acoustic features (low-frequency energy ratio and nocturnal snoring frequency) between the cerebrovascular stenosis and non-stenosis groups. These findings provide preliminary evidence that snoring acoustics may serve as potential indicators of cerebrovascular risk, although further validation in larger cohorts is required.

### 4.1 Clinical significance and application scenarios

Based on the current findings, the proposed method demonstrates potential clinical application value in three aspects. First, in community or outpatient screening settings, automated snoring analysis can serve as a low-cost, non-invasive auxiliary tool for the preliminary identification of suspected cases. For example, patients exhibiting a markedly elevated nocturnal snoring frequency (≥800 events during an 8-h sleep period) and a clearly reduced low-frequency energy ratio could be prioritized for carotid ultrasound or other vascular assessments. Second, the model maintained stable performance under noisy conditions, indicating its applicability in hospital wards and home sleep monitoring scenarios. This adaptability expands the potential for continuous and long-term observation of high-risk individuals outside laboratory environments. Third, the observed acoustic differences between the stenosis and non-stenosis groups suggest that snoring features may serve as early warning indicators of cerebrovascular abnormalities in patients with OSAHS and help clinicians identify which patients require further vascular evaluation.

Future studies should establish quantitative criteria for referral or further evaluation, such as defining what level of nocturnal snoring frequency or range of low-frequency energy ratios should be considered clinically significant. These criteria should be validated through larger, prospective cohort studies.

### 4.2 Limitations and future work

Despite the advantages demonstrated by the proposed method, several limitations remain. First, the sample size is limited to only 31 patients, leading to insufficient representativeness that may compromise the generalizability of the findings. Future studies should expand into multicenter settings and include participants across different age groups. Second, the method relies on two-dimensional spectrograms and convolutional neural networks, which, although effective in capturing time-frequency features, entail relatively high computational costs. Previous studies have proposed snoring classification methods based on one-dimensional features, such as amplitude spectrum trend features (Sun et al., 2020) or representation learning based on auditory receptive fields (Hu et al., 2023). These approaches generally offer higher computational efficiency and deployment convenience but remain inadequate for modeling complex time-frequency patterns. Future research could explore integrating two-dimensional spectral analysis with one-dimensional feature extraction to balance discriminative power and computational efficiency. Finally, the acoustic differences identified in this study are based solely on cross-sectional statistical analysis, making it difficult to directly infer the underlying pathological mechanisms. Larger-scale longitudinal studies are needed to validate their clinical significance.

## 5 Conclusion

This paper proposes a snoring classification method integrating dynamic convolution with attention mechanisms, with a particular emphasis on exploring the acoustic differences between patients with and without cerebrovascular stenosis. The main conclusions and contributions are as follows: (1) Methodological improvements: Among four acoustic features and multiple baseline models, this paper proposes integrating AKConv, CBAM, and Conv2Former modules into the ConvNeXt backbone. On the Mel spectrogram, the method achieves 90.24% accuracy in classifying snoring *versus* non-snoring sounds, representing an improvement of approximately 2 percentage points over the ConvNeXt baseline. It also achieves 84.68% accuracy in classifying stenotic *versus* non-stenotic snoring sounds, which should be regarded as preliminary. (2) Robustness and ablation validation: Through noise robustness experiments, the proposed method was shown to maintain stable performance under varying noise conditions. Furthermore, ablation studies confirm that each added module contributes incrementally to the observed improvements, underscoring the reliability and interpretability of the proposed architecture. (3) Preliminary clinical insights: Clinical analyses identified significant differences between stenotic and non-stenotic patients in low-frequency energy ratio (p = 0.025) and nocturnal snoring frequency (p = 0.031). Sensitivity analyses excluding patients with cerebral infarction and cerebral ischemia yielded consistent results (p = 0.038 and p = 0.018, respectively). Snoring event duration did not differ significantly. These clinical-statistical findings are promising but remain preliminary and require validation in larger-scale, longitudinal studies before any clinical application.

## Data Availability

The raw data supporting the conclusions of this article will be made available by the authors, without undue reservation.
