# Supplementary material for: Snoring sound classification in patients with cerebrovascular stenosis based on an improved ConvNeXt model
Source: Front Physiol. 2025 Nov 26;16:1661258. doi: 10.3389/fphys.2025.1661258 (PMC12689313; doi:10.3389/fphys.2025.1661258)
Supplement: Supplementary file 1 [file DataSheet1.pdf]

# Supplementary Material

## 1 SUPPLEMENTARY TABLES AND FIGURES

### 1.1 Determination of $\beta$ via Validation Grid Search

To determine the optimal enhancement factor $\beta$, we performed a grid search on the ConvNeXt-T baseline validation set with candidates $\beta \in 1.1, 1.2, \ldots, 2.0$ (step 0.1). Each candidate was evaluated by repeating the training three times and recording the validation F1-score; results are reported as mean ± SD in Table S1. Both Mel and CQT spectrograms exhibit peak performance near $\beta = 1.5$ (Mel: $88.07\% \pm 0.08$, CQT: $86.09\% \pm 0.16$). The selected value $\beta = 1.5$ was then applied consistently in all subsequent experiments.

**Table S1.** Grid-search results of $\beta$ on the ConvNeXt-T validation set. Values are reported as mean ± SD over three runs.

| $\beta$ | Mel spectrogram (F1-score, %) | CQT spectrogram (F1-score, %) |
|---|---|---|
| 1.1 | 87.50 ± 0.05 | 85.45 ± 0.05 |
| 1.2 | 87.72 ± 0.02 | 85.73 ± 0.04 |
| 1.3 | 87.90 ± 0.04 | 85.90 ± 0.04 |
| 1.4 | 88.03 ± 0.09 | 86.05 ± 0.05 |
| 1.5 | **88.07** ± **0.08** | **86.09** ± **0.16** |
| 1.6 | 88.01 ± 0.10 | 86.05 ± 0.05 |
| 1.7 | 87.82 ± 0.07 | 85.90 ± 0.05 |
| 1.8 | 87.66 ± 0.05 | 85.67 ± 0.08 |
| 1.9 | 87.40 ± 0.05 | 85.37 ± 0.08 |
| 2.0 | 87.10 ± 0.05 | 85.05 ± 0.03 |

### 1.2 Patient-wise 5-Fold Cross-Validation Data Distribution

To evaluate the proposed method performance on snoring versus non-snoring classification while avoiding inter-subject data leakage, a 5-fold patient-wise cross-validation was conducted. The dataset from 31 patients was partitioned into five folds, with each fold containing data from distinct patients to ensure independence between folds. In each iteration, one fold was used as the validation set, and the remaining four folds served as the training set. This procedure was repeated five times so that each fold acted as the validation set exactly once.

Table S2 presents the patient-wise 5-fold distribution of snoring and non-snoring segments for stenotic and non-stenotic patients. The number of segments varies across folds due to differences in the amount of data contributed by each patient. The total counts across all folds correspond to the entire dataset.

### 1.3 Patient-level Aggregated Performance

In addition to the segment-level results reported in the main text (Tables 3 and 4), model performance was also evaluated at the patient level. For this analysis, each validation patient was assigned a single predicted label based on majority voting across all their segments. Patient-level labels were derived by aggregating segment-level predictions, and evaluation metrics were calculated using standard definitions (e.g., sensitivity based on positive patients, specificity based on negative patients). Metrics were expressed

**Table S2.** Distribution of snoring and non-snoring segments within each fold

| Fold | Snoring | | Snoring Total | Non-snoring | | Non-snoring Total | Total |
|---|---|---|---|---|---|---|---|
| | Stenotic | Non-stenotic | | Stenotic | Non-stenotic | | |
| Fold 1 | 4001 | 1982 | 5983 | 3950 | 2315 | 6265 | 12248 |
| Fold 2 | 3987 | 1970 | 5957 | 3985 | 2300 | 6285 | 12242 |
| Fold 3 | 4065 | 1930 | 5995 | 3990 | 2315 | 6305 | 12300 |
| Fold 4 | 4040 | 1985 | 6025 | 3955 | 2303 | 6258 | 12283 |
| Fold 5 | 4075 | 2010 | 6085 | 3974 | 2303 | 6277 | 12362 |
| **Total** | 20168 | 9877 | 30045 | 19854 | 11536 | 31390 | 61435 |

as n/N (percentage), where n is the number of patients correctly classified for a given metric and N is the total number of patients relevant to that metric (e.g., positive patients for sensitivity, negative patients for specificity). The F1-score was computed from sensitivity and PPV. As shown in Table S3 and Table S4, the proposed method consistently outperformed the ConvNeXt baseline at the patient level for both snoring vs. non-snoring and stenotic vs. non-stenotic classification tasks.

**Table S3.** Patient-level aggregated performance for snoring vs. non-snoring classification (n = 6 validation patients).

| **Model** | **Accuracy** *n/N (%)* | **Sensitivity** *n/N (%)* | **Specificity** *n/N (%)* | **PPV** *n/N (%)* | **F1-score** *(%)* |
|---|---|---|---|---|---|
| ConvNeXt-T | 5/6 (83.33) | 4/5 (80.00) | 1/1 (100.00) | 4/5 (80.00) | 80.00 |
| This paper | 6/6 (100.00) | 5/5 (100.00) | 1/1 (100.00) | 5/5 (100.00) | 100.00 |

Note: Patient-level metrics are reported as n/N (percentage), where n is the number of patients correctly classified for the given metric and N is the total number of patients relevant to that metric (e.g., positive patients for sensitivity, negative patients for specificity). F1-score was derived from sensitivity and PPV.

**Table S4.** Patient-level aggregated performance for stenotic vs. non-stenotic snoring classification (n = 6 validation patients).

| **Model** | **Accuracy** *n/N (%)* | **Sensitivity** *n/N (%)* | **Specificity** *n/N (%)* | **PPV** *n/N (%)* | **F1-score** *(%)* |
|---|---|---|---|---|---|
| ConvNeXt-T | 4/6 (66.67) | 3/4 (75.00) | 1/2 (50.00) | 3/5 (60.00) | 66.67 |
| This paper | 5/6 (83.33) | 4/4 (100.00) | 1/2 (50.00) | 4/5 (80.00) | 88.89 |

Note: Patient-level metrics are reported as n/N (percentage), where n is the number of patients correctly classified for the given metric and N is the total number of patients relevant to that metric (e.g., positive patients for sensitivity, negative patients for specificity). F1-score was derived from sensitivity and PPV.
